# Designs, facilitators, barriers, and lessons learned during the implementation of emergency department led virtual urgent care programs in Ontario, Canada

**DOI:** 10.3389/fdgth.2022.946734

**Published:** 2022-08-24

**Authors:** Justin N. Hall, Alun D. Ackery, Katie N. Dainty, Paul S. Gill, Rodrick Lim, Sameer Masood, Shelley L. McLeod, Shaun D. Mehta, Larry Nijmeh, Daniel Rosenfield, Greg Rutledge, Aikta Verma, Shawn Mondoux

**Affiliations:** ^1^ Department of Emergency Services, Sunnybrook Health Sciences Centre, Toronto, ON, Canada; ^2^ Division of Emergency Medicine, Department of Medicine, University of Toronto, Toronto, ON, Canada; ^3^ Department of Emergency Medicine, St. Michael’s Hospital, Unity Health, Toronto, ON, Canada; ^4^ Department of Research and Innovation, North York General Hospital, Toronto, ON, Canada; ^5^ Institute of Health Policy, Management and Evaluation, University of Toronto, Toronto, ON, Canada; ^6^ Department of Community and Family Medicine, University of Toronto, Toronto, ON, Canada; ^7^ Department of Paediatrics and Medicine, Western University, London, ON, Canada; ^8^ Department of Emergency Services, University Health Network, Toronto, ON, Canada; ^9^ Schwartz/Reisman Emergency Medicine Institute (SREMI), Mount Sinai Hospital, Toronto, ON, Canada; ^10^ Department of Emergency Medicine, Lakeridge Health, Oshawa, ON, Canada; ^11^ Department of Family Medicine, Queen’s University, Kingston, ON, Canada; ^12^ Division of Paediatric Emergency Medicine, The Hospital for Sick Children, Toronto, ON, Canada; ^13^ Department of Paediatrics, University of Toronto, Toronto, ON, Canada; ^14^ Department of Emergency Medicine, St. Joseph’s Healthcare Hamilton, Hamilton, ON, Canada; ^15^ Division of Emergency Medicine, Department of Medicine, McMaster University, Hamilton, ON, Canada

**Keywords:** virtual care, emergency services, urgent care, COVID-19, implementation

## Abstract

**Introduction:**

Virtual patient care has seen incredible growth since the beginning of the COVID-19 pandemic. To provide greater access to safe and timely urgent care, in the fall of 2020, the Ministry of Health introduced a pilot program of 14 virtual urgent care (VUC) initiatives across the province of Ontario. The objective of this paper was to describe the overall design, facilitators, barriers, and lessons learned during the implementation of seven emergency department (ED) led VUC pilot programs in Ontario, Canada.

**Methods:**

We assembled an expert panel of 13 emergency medicine physicians and researchers with experience leading and implementing local VUC programs. Each VUC program lead was asked to describe their local pilot program, share common facilitators and barriers to adoption of VUC services, and summarize lessons learned for future VUC design and development.

**Results:**

Models of care interventions varied across VUC pilot programs related to triage, staffing, technology, and physician remuneration. Common facilitators included local champions to guide program delivery, provincial funding support, and multi-modal marketing and promotions. Common barriers included behaviour change strategies to support adoption of a new service, access to high-quality information technology to support new workflow models that consider privacy, risk, and legal perspectives, and standardized data collection which underpin overall objective impact assessments.

**Conclusions:**

These pilot programs were rapidly implemented to support safe access to care and ED diversion of patients with low acuity issues during the COVID-19 pandemic. The heterogeneity of program implementation respects local autonomy yet may present challenges for sustainability efforts and future funding considerations.

## Introduction

Prior to the COVID-19 global pandemic, the Ontario Ministry of Health released their “Digital First for Health” report in November 2019 as part of a strategy to end “hallway medicine” and address overcrowding in Ontario hospitals. The five pillars of this strategy included more virtual care options, expanded access to online appointment booking, greater patient access to personal health information, better and more connected tools for frontline providers, data integration and predictive analytics (1). In support of this work in early 2020, the Ontario Telemedicine Network announced their goal to provide local virtual access to Emergency Services (2). When the global pandemic was announced in March 2020, many aspects of the Ontario healthcare system were slowly beginning to offer more virtual care services (3, 4). Virtual care services include “any interaction between patients and/or members of their circle of care, occurring remotely, using any forms of communication or information technologies, with the aim of facilitating or maximizing the quality and effectiveness of patient care” (5). Throughout the pandemic, virtual patient care has seen incredible growth and rapid acceleration (6).

Prior to the COVID-19 pandemic, there was a paucity of data related to virtual urgent and emergent care services in the published literature (7, 8). As a result of innovations stemming from the pandemic, additional VUC services have been described for multiple American, Canadian, and Australian programs (9–14). Despite the recent surge in publications related to virtual urgent and emergent care, implementation lessons learned remain underreported (14).

To provide greater access to safe and timely urgent care, in the fall of 2020, the Ministry of Health introduced a pilot program of 14 emergency department (ED) led virtual urgent care (VUC) initiatives across the province of Ontario, the most populous province in Canada, to enable healthcare providers and patients to safely connect from a distance (15). These programs were overseen by the Virtual Care Secretariat at Ontario Health, a provincial healthcare administrative agency, and were commissioned to provide VUC services to five Ontario Health regions (Toronto, Central, East, West, and North) for a minimum of 6 months. While the design and model delivery of each pilot program varied, the overall aim was to support ED diversion of patients with low acuity issues and reduce the need for face-to-face contact whenever possible, limiting potential COVID-19 exposure. The objective of this paper was to describe the overall design, facilitators, barriers, and lessons learned during the implementation of seven ED led VUC pilot programs in Ontario, Canada.

## Methods

### Application and approval process

Hospital organizations interested in establishing a VUC service submitted a funding application to Ontario Health with their proposed pilot program design to address local community needs. Each proposal was reviewed by the local Ontario Health region and the provincial Virtual Care Secretariat in an iterative manner. Feedback was shared with the pilot program leads regarding proposed pilot program design and implementation considerations related to triage, staffing model, technology use, program leadership, and hours of service. Each application underwent two or three rounds of consideration by Ontario Health prior to funding approval. Programs were expected to launch their services within one to two months of funding approval to help alleviate healthcare access pressures secondary to the global pandemic.

### Iterative local pilot program design and evaluation

Sites were responsible for designing and implementing their VUC programs in accordance with the overall funding program objectives. There were no specific directives given by Ontario Health or the Ministry of Health in terms of VUC program design specifications. As a result, the clinical and operational governance, technology, staffing, patient engagement, and VUC services offered varied based on the specific use case of each local institution.

As part of the conditions to receive provincial program funding, sites were required to contribute to a provincial evaluation including monthly secure data transfer of standardized reporting metrics related to patient presentations and reasons for use, volumes, and patient-reported outcomes and attend monthly provincial evaluation meetings. Each program signed a service agreement which outlined program deliverables (monthly data reporting, confirmation of approved technology vendor of record for documenting patient visits, and lessons learned report) and patient volume targets to receive their full funding allocation. Beyond these minimum requirements, each site was encouraged to evaluate their own local pilot program, iterate, and enhance their VUC services based on patient and provider feedback. Regular discussions across pilot sites afforded the opportunity to leverage shared learnings, innovations, and sustainability ideas.

### Data collection from VUC site leads

We assembled an expert panel of 13 emergency medicine physicians and researchers with experience leading and implementing VUC programs. This diverse group represented healthcare providers and researchers from pediatric, urban, regional, academic and community sites across the province. The current work describes seven of the fourteen pilot program sites. Each VUC program lead was asked to complete a standardized data collection template to provide an overview of their VUC model and infrastructure, share common facilitators and barriers to adoption of VUC services, and summarize lessons learned for future VUC design and development.

The two study leads (JNH and SM) reviewed these site-specific descriptions and learnings and prepared summaries of key themes for pediatric, urban, and regional programs. One study author then facilitated discussion with local program leaders for each of these three areas to ensure the summaries reflected their shared experiences. Feedback was incorporated into revised summaries until consensus was achieved. An overall summary including the most common facilitators, barriers, and lessons learned was reviewed and agreed upon by the study team.

### Impact of VUC services

While not a barrier to individual program implementation or evaluation, the rapid asynchronous launch of programs and the lack of standardized data collection tools at the provincial level has presented challenges to assessing overall impact across the pilot program. Initially, each site was responsible for their own evaluation plans, with some sites adopting rigorous research agendas while others focused on local quality improvement efforts or did not have a formal evaluation plan at the outset. As the number and diversity of pilot projects approved for provincial funding increased, it became apparent that a coordinated provincial evaluation plan would be necessary to ensure responsible stewardship of limited resources. A provincial steering committee to represent research, policy and clinical perspectives and guide the evaluation efforts was formed. A larger provincial evaluation committee with representation from each of the VUC pilot sites, Ontario Health, and the Ontario Ministry of Health was also established. Common data reporting requirements were enacted for pilot sites to receive full funding. These committees met regularly to analyze data, discuss current data trends, share learnings and challenges, and develop plans for sustainability.

## Results

### VUC services

Models of care varied across VUC pilot programs. Several of the pilot sites adopted a collaborative approach with primary care, long-term care facilities, and community partners. These collaborations generally involved increased awareness of the VUC services, referral pathways for their clients, and access to technology (e.g., facilitating use of a phone or computer through a community organization). A summary of each of the VUC pilot programs is displayed in Table 1. Key areas of variation related to triage, staffing, technology, and physician remuneration are discussed in more detail below.

**Table 1:** Virtual urgent care pilot program models.

Program	Program #1	Program #2	Program #3	Program #4	Program #5	Program #6	Program #7
Sign-up (online/phone/both)	Both	Both	Both	Both	Both	Both	Online
Advertisement modalities	Twitter, Facebook, Hospital website, Traditional media, Signage and business cards in EDs and vaccine clinics	Google Ad Words, Instagram, Facebook, Twitter, Hospital website	Twitter, Instagram, Facebook, In-hospital signage, Embedded in “After Visit Summary” (discharge instructions)	Google Ad Words, Instagram, Facebook, Twitter, Hospital website	Hospital website, Facebook, Instagram, Twitter, Traditional media, In-hospital signage, Discharge postcards	Hospital website, Facebook, Twitter, Patient portal, ED signage, Fliers with QR codes	Hospital website, Twitter, Instagram, Traditional media
EHR use (Y/N)	Y	Y	Y	Y	Y	Y	Y
EHR name	Corolar Virtual Care (CVC app integrated with Teams)	Cerner	Epic	Epic	SunnyCare	Quadramed	Soarian Clinicals (Cerner)
HCP triage (Y/N)	Y	Y	N	Y	N	Y	N
eCTAS (Y/N)	N	Y	N	N	N	N	N
Designation of triage provider (e.g., RN, MD, N/A)	RN	RN, MD	N/A	RN	N/A	NP	N/A
Scheduling platform (name)	CVC Virtual Waiting Room	Verto	Digital Waiting Room	Epic	Inhouse platform	Inhouse platform	Verto
Care provider interaction (video vs phone vs both)	Both	Both	Video	Both	Video	Both	Both
Video platform (Zoom®, Microsoft® Teams, other)	Microsoft Teams	WebEx	Other	Zoom Healthcare	Zoom Healthcare	Microsoft Teams	Zoom Healthcare
Visit record available in database within 24 h (Y/N)	Y	Y	Y	Y	Y	Y	Y
Virtual visit care by MD (Y/N and % of coverage)	Y, 100%	Y, 100%	Y, 100% (reviewed)	Y, 100%	Y, 100%	Y, 95%	Y, 100%
Virtual visit care by alternate provider (designations and % of coverage)	N/A	N/A	PA, 100%	N/A	N/A	NP, 5%	N/A
Hours of virtual care availability per day of week [weekday (M-F), weekend (S/S), holiday (H)]	M-F, 9AM-6PM	M-F, 12-8PM	M-F, 9AM-1AM	M-F, 12-8PM	M-F, 2-9PM	M-F, 2-9PM	M-F, 2-9PM
	S/S, 10AM-3PM	S/S, 12-8PM	S/S, 9AM-1AM	S/S, 12-8PM	S/S, 0	S/S, 0	S/S, 0
	H, 10AM-3PM	H, 12-8PM	H, 9AM-1AM	H, 12-8PM	H, 2-9PM	H, 2-9PM	H, 0
Hours of virtual care availability per day of week after 5PM	1	3	8	3	4	4	4
Access to community imaging or lab orders (Y/N)	Y	N	N	N	N	N	N
Access to elective hospital imaging (Y/N)	Y	N	N	N	Y	Y	Y
Patient access to medical record (Y/N)	Y	Y	Y	Y	Y	Y	Y
Service can be provided by the practitioner when working outside of the hospital (Y/N)	Y	Y	Y	Y	Y	Y	Y
Service volume (Dec 2020–Sept 2021)^a^	3,024	3,301	744	2,271	1,567	1,445	642
Program extended beyond initial 6-month pilot	Y	Y	Y	Y	Y	Y	Y

EHR, electronic health record; HCP, health care provider; eCTAS, Electronic Canadian Triage and Acuity Scale.

^a^ Highly variable launch dates from December to April with some sites operational before this funding program commenced.

#### Triage models

Triage models included self-screening, nurse-led triage, and physician-driven triage. Three of the VUC pilot sites used a self-screening model, where patients were asked to review recommended indications for a VUC visit and select their primary complaint when they registered. An advantage of this system was that patients described their history once, thus reducing redundancy and the amount of time required for the patient interaction; the disadvantage was that a patient may wait in queue until their scheduled appointment despite having more urgent symptoms than another patient scheduled before them. However, no patient was declined an appointment based on their reported concerns. It was felt that patients who had symptoms likely to need an in-person assessment (e.g., chest pain) but chose a VUC consultation would benefit from a discussion with the virtual ED physician about the benefits and risks of attending the ED in-person. Furthermore, it was helpful for the virtual ED physician to perform an assessment, write a detailed note and facilitate a “warm hand-off” to the in-person ED physician to streamline patient care (e.g., pre-empt investigations, reduce redundancies in assessment). Other programs preferred a care model with a triage nurse (3, 1 of which also used a team decision model with a physician) or nurse practitioner (1) as the first point of contact to ensure patients with acute illness were diverted to the most appropriate level of care. It was acknowledged that without a triage nurse service, patients with significant acute illness may wait in queue to be seen in an inappropriate care setting. The downsides of this model were that patients were required to discuss their concern twice, requiring additional time for their consultation, and the human resource costs were higher to include a triage nurse/nurse practitioner.

#### Staffing models

Staffing within the models varied for both clinicians and administrators. Staff were recruited at most sites on a voluntarily basis from existing providers and administrators working within the hospitals. Program leaders identified several distinct advantages of voluntary, rather than mandatory, participation: provider openness to innovate and contribute to system-level change to develop a new model of care, enthusiasm despite initially greater uncertainty related to care pathways and the types of patients that would use the service, and intrinsic motivation to help patients access care who otherwise may not have accessed healthcare services in a timely manner. Recruitment was more challenging at some sites as this was considered an “extra” ask above and beyond one's normal clinical hours, with some hospitals already mandating extra in-person work hours due to human resource challenges and heightened patient volumes because of the pandemic.

Some programs were exclusively staffed by physicians (emergency medicine and/or general practitioner), while others involved a nurse, physician assistant, or nurse practitioner as an intermediate step between the patient and physician. Clinicians were located within the hospital for some models, while others afforded the flexibility to complete shifts from outside the hospital (i.e., connecting from home or an off-site clinic location) to help address local human resource needs related to quarantine/isolation, family considerations, sick calls, and limiting potential COVID-19 exposure for some senior or immunocompromised physicians.

Some of the VUC pilot programs were staffed by on-call emergency medicine physicians responsible for other tasks (e.g., imaging discrepancies, lab/urine follow ups) who could work remotely. As part of a separate initiative, some sites provided a call-in number to community general practitioners to discuss hospital resources, provide peer-to-peer consultations, and arrange assessment for any patient in the community. One of the paediatric VUC sites utilized physician assistants in addition to pediatric emergency medicine specialists for virtual care. Registration clerks were also identified as vital to providing VUC services. They assisted with faxing of notes of patient visits to peripheral EDs, clinics, or referrals and coordinating next steps in the patient's journey.

#### Technology platforms

The technology used to provide the virtual care programs varied across sites as well, as there is no single provincial electronic health record (EHR) nor a common virtual front door for urgent care services in Ontario. All but one site used the same EHR system to document patient encounters for VUC as they use in their in-person EDs. All sites also built a dedicated VUC website (some also had dedicated phone lines), and most sites needed to purchase or build an online booking system for patients to access VUC services (see Table 1). The booking system platforms allowed sites to capture patient demographics, reasons for using the service, and brief clinical presentation information to support triage, registration, and data collection. Some allowed for automated SMS text message and email confirmations, appointment reminders, and follow-up patient feedback surveys to be sent, while others required administrative support for these activities. The level of access and comfort of using technology for patients varied widely based on the user-friendliness of the platforms and patient and provider demographics.

#### Physician remuneration

Ontario operates a single payer system through which physicians are remunerated based on a standardized provincial fee schedule for services rendered. Shortly after the global pandemic was declared, temporary physician billing codes were established by the Ontario Ministry of Health for physicians providing virtual care services. Although all VUC pilot sites billed in a fee-for-service mode for patient assessments, physician remuneration varied across programs based on patient volumes, model and hours of coverage, and other simultaneous physician responsibilities.

Additionally, emergency medicine physicians working in some EDs are compensated through an Alternative Funding Agreement (AFA) (16) which provides EDs with a set number of hours of coverage based on patient volumes and acuity scores from the previous year, while simultaneously recognizing their teaching commitments for medical student and resident physician learners. For AFA sites, there was a potential conflict between the current physician remuneration model for VUC services and for in-person ED care. If efforts to divert patients from the physical ED to VUC were successful, ED volumes would be reduced, resulting in less funded ED physician coverage hours for the subsequent year as VUC visits are not currently included in overall ED volumes.

### Adoption of VUC services

#### General facilitators

Several facilitators to adoption of VUC services were experienced similarly by the pilot sites and are displayed in Table 2. Beyond these, many of the sites said a main facilitator to adoption of VUC services at this time was the “burning platform” for alternative care options during the pandemic. There were patients with concerning symptoms that needed to seek care in a physical ED but were afraid to attend in-person; speaking to a VUC physician helped alleviate their fears and confirmed that a physical ED visit was necessary and worth any perceived risk or exposure. There were also patients with relatively minor problems, who perceived great value in learning an in-person ED visit would not be necessary, as alternative options could be discussed. Additionally, from a provider perspective, ED physicians appreciated the opportunity to speak to their patients in a calm and relaxed environment, a welcomed change from the taxing and chaotic grind of the physical ED during a pandemic.

**Table 2:** Most common facilitators for implementation.

• Identifying a local champion/program lead to guide efforts and build clinician citizenship (buy-in)	• Having visionary leaders/administrators who support and foster change management
• Securing access to provincial funding and fair physician remuneration	• Addressing patient fears related to attending the ED in-person
• Incorporating patient perspectives throughout the planning process and seeking active feedback through coordinated post-encounter surveys	• Providing options for clinicians to complete the shifts both inside and outside the hospital
• Creating patient awareness through multi-modal marketing and varied communications	• Collaborating with IT departments skilled in digital transformation

There were some additional facilitators for VUC identified specifically by the paediatric sites. For example, most parents of young children are more technologically savvy than older adults and are less intimidated/more familiar with platforms such as Zoom®, Microsoft® Teams and others and more comfortable with online interaction. They are also more likely to own smartphones, laptops, and other electronic devices, which seemed to support better program uptake.

#### General barriers

Several barriers to adoption of VUC services were shared across multiple sites and are displayed in Table 3. One of the main barriers to better uptake of the VUC programs was public awareness. For anyone who used VUC services (both patient and provider), satisfaction appeared to be high and almost all would recommend to others [see McLeod et al. (17) for complete descriptive analysis of patient characteristics and site-by-site comparison of programs]. However, initial enrollment into many of the VUC programs was less than anticipated. Behaviour change strategies to support adoption of these new services were implemented at multiple sites. These included public education through in-ED signage, pamphlets, and discussions with frontline clerical and clinical staff for follow-up of non-emergent issues, community-driven communication from local healthcare providers, and targeted messaging regarding the benefits of using VUC services (e.g., timely appointments, ease of using the service, reduced face-to-face contact and potential COVID-19 exposure, saved travel time and costs). After implementing multi-modal communication and advertisement strategies, most sites offering VUC services were still not fully booked on a consistent basis.

**Table 3:** Most common barriers for implementation.

• Developing new workflow models including privacy, legal, ethical, and risk considerations	• Changing behaviours inside and outside the hospital environment to raise awareness and encourage uptake of the new service
• Ensuring the program resonated with diverse patient populations while considering language and socioeconomic barriers	• Accessing high-quality technology and system integration for patients and providers (high-speed Internet, cameras, unified EHR, e-faxing prescriptions)
• Communicating with patients during a pandemic to raise awareness for the new service	• Promoting standardized data collection across sites to support overall objective impact assessments

Many of the VUC sites stated digital inequity as a barrier to VUC. Some patients who tend to use the ED for low-acuity problems would be well-suited to use VUC services, but they do not have access to a smartphone or computer. One urban site tried to bridge this gap by donating IT infrastructure to local shelters and recruiting the shelter staff to facilitate clients’ appointments. In addition, lack of a unifying electronic health record and the need for data sharing agreements and research ethics board approval across institutions presented additional challenges.

### Facilitators and barriers by model type

#### Triage models

While nurse triage is standard practice for EDs, sites that developed self or physician triage models benefited from having patient perspectives incorporated through the planning and implementation phases as patients were the primary drivers toward developing these alternative screening options to reduce redundancy and save time. Moreover, these models were more likely to be successful when visionary leaders/administrators supported and fostered the change management process given the new workflow models, including privacy, legal, ethical, and risk considerations, that had to be developed. Two main barriers to nurse triage were the availability of sufficient triage nurses given staffing challenges within Ontario EDs and the inability to determine an accurate Canadian Triage and Acuity Scale (CTAS) score for all patients given limited access to vital sign parameters due to the virtual nature of the service.

#### Staffing models

Independent of the type of frontline clinicians engaged in the programs, identifying a local champion/program lead to guide efforts and build clinician citizenship (buy-in) was invaluable toward both staffing recruitment and retention efforts. Moreover, programs that allowed flexibility in staffing location and specifically offered remote options fostered clinician uptake, supported clinician wellness, and allowed those requiring in-person isolation due to close COVID-19 contacts to still complete shifts. Finally, those clinicians who did not have competing responsibilities at the same time described their experience more favourably than those who had other simultaneous clinical responsibilities.

#### Technology platforms

Patients and clinicians described several facilitators related to technology. Systems that allowed for automated SMS text message and email confirmations, appointment reminders, and follow-up patient feedback surveys to be sent were rated highly as they were more efficient for administrators and supported personalized messages using the patient's preferred communication modality. Programs that collaborated with their information technology/information services departments to develop automated systems were more nimble and better supported the standardized data collection and monthly submission to the funder than those that adopted manual data processes. This also enhanced near real-time awareness of program uptake and patient satisfaction to help support further marketing and communication efforts. Two main technology barriers cited by some programs were not having access to a unified electronic health record to gain a full understanding of a patient's medical history, and the inability to e-fax prescriptions and assessment notes directly to a patient's circle of care (rather they had to manually fax with the support of administrative personnel).

## Discussion

We describe the variation in overall design, facilitators, barriers, and lessons learned during the implementation of seven ED led VUC pilot programs in Ontario, Canada. Models of care interventions varied across the VUC pilot programs related to triage, staffing, technology, and physician remuneration. Common facilitators included local champions to guide program delivery, provincial funding support, incorporating patients throughout the planning process, and multipronged approaches to marketing and promotions. Common barriers included behaviour change strategies to support adoption of a new service, access to high-quality information technology to support new workflow models that consider privacy, risk, and legal perspectives, strategies to ensure equitable access for patients independent of socioeconomic background, and standardized data collection to support overall objective impact assessments.

Moreover, the current work addresses an important gap in the published literature related to VUC implementation strategies and lessons learned. Prior to the COVID-19 pandemic, there was a paucity of data related to virtual urgent and emergent care services in the published literature (7, 8). As a result of innovations stemming from the pandemic, additional VUC services have been described for multiple American, Canadian, and Australian programs (9–14). Despite the recent surge in publications related to virtual urgent and emergent care, implementation lessons learned remain underreported (14).

We identify several important lessons learned regarding implementation of VUC services and the challenges of rapid, real-time implementation. First, local autonomy in program design, development, implementation, and iterative improvements is a double-edged sword. Autonomy empowers programs to reflect the needs of local communities, fosters engagement, and affords much opportunity to study a myriad of approaches. Yet this flexibility creates multiple challenges to directly compare programs, pool data, determine overall impact, and identify recommendations for future funding. While the VUC pilot programs described in the current work were rapidly introduced, many incorporated standard elements of program design such as a leadership structure, budget, patient engagement, and evaluation plan, and thus were not victim to many of the shortcomings recently described by a similar program implementation in Australia where rapid cycling and implementation were at the expense of evaluation and sustainability efforts (14).

Second, although similar design and implementation strategies were utilized across VUC programs, how the various program components integrated to create a seamless patient experience influenced uptake and sustainability. Models that had a local program champion, strong clinician citizenship, supportive hospital leaders/administrators, integrated patient perspectives through the design, implementation, and evaluation phases, and created broad patient awareness through multimodal communications and marketing saw higher patient volumes (see Table 1). Given the ongoing waves of the COVID-19 pandemic and need for timely access to care, an additional funding call was launched whereby programs that had met their service agreement deliverables and patient volume targets were able to apply for additional program funding, with all the programs described in this paper extending their programs beyond the initial 6-month pilot.

Third, virtual care has been previously shown to improve access to care and reduce health inequities among marginalized populations (18–20). However, the current work found access to technology as a barrier to participation for some patients with low-acuity health concerns within the urban VUC programs. Despite being well-suited to use VUC services, some marginalized and underhoused patients did not have access to a smartphone or computer, and therefore coordination with local community organizations and health agencies to help facilitate access was essential. It is important to be mindful of this barrier when any digital solution is implemented as it may create inherent bias with respect to whom it serves.

Finally, early program results suggest funding models can be leveraged to bring disparate organizations together toward regional and provincial goals for overall VUC improvement and better patient experience. While one regional program leveraged a shared digital front door from the outset to capitalize on pediatric, adult, and long-term care expertise, another region has adopted this approach as part of their iterative improvements to offer patients a more seamless experience and enhance resource sharing for program efficiency. Moreover, one VUC program has successfully incorporated some of the larger regional primary care clinics in their model to see both rostered (those with a primary care provider) and unattached patients (those without a primary care provider) who have registered.

### Future directions

Several priority considerations remain as we look to the future of VUC: iterating program offerings based on emerging evidence to support continuous quality improvement; continuing our robust multi-modal evaluation both locally and provincially; developing a community of practice related to virtual emergency services; engaging in sustainability planning based on shared learnings; integrating primary care as part of a “primary care first” strategy with the opportunity to escalate to ED physicians who can serve as a conduit for hospital-based services; and influencing health policy and ongoing funding decisions to include VUC services as part of broader health system transformation to ensure patients are able to access the right care at the right time in the right place.

### Limitations

The first limitation of the current work is that not all pilot programs chose to participate in academic evaluative collaborations beyond the minimum requirements of the program, and therefore, the current facilitators, barriers, and lessons learned may not be representative of all pilot VUC programs. The second limitation is the heterogeneity in program implementation makes monitoring the degree of fidelity and effectiveness of this funding very challenging. Standardized data collection from the outset of program planning and implementation is critical to support overall objective impact assessments.

## Conclusion

These VUC pilot programs in Ontario Canada were rapidly implemented to support safe access to care and ED diversion of patients with low acuity issues during the COVID-19 pandemic. The heterogeneity of program implementation respects local autonomy yet may present challenges for guiding sustainability efforts and future funding considerations.

## Data Availability

The original contributions presented in the study are included in the article/Supplementary Material, further inquiries can be directed to the corresponding author.
